# Activation of Necroptosis by Engineered Self Tumor-Derived Exosomes Loaded with CRISPR/Cas9

**DOI:** 10.1016/j.omtn.2019.05.032

**Published:** 2019-07-03

**Authors:** Diana Gulei, Ioana Berindan-Neagoe

**Affiliations:** 1 MEDFUTURE - Research Center for Advanced Medicine, Iuliu Hatieganu University of Medicine and Pharmacy, Marinescu 23 Street, 400337 Cluj-Napoca, Romania; 2 Research Center for Functional Genomics, Biomedicine and Translational Medicine, Iuliu Hatieganu University of Medicine and Pharmacy, Marinescu 23 Street, 400337 Cluj-Napoca, Romania; 3 Department of Functional Genomics and Experimental Pathology, The Oncology Institute Prof. Dr. Ion Chiricuta, Republicii 34-36 Street, Cluj-Napoca, Romania

**Keywords:** exosomes, CRISPR/Cas9, necroptosis, cancer, therapy

## Abstract

CRISPR/Cas9 has proved its efficiency *in vitro*, where we now know that this tool can efficiently target specific parts of the genome. These modifications can be used to generate advanced models of human diseases, address specific functions of genes, and develop new therapeutic strategies. Even if these advancements are promising, there are still two great issues associated with CRISPR/Cas9: how we can specifically and safely deliver the editing tool *in vivo* and how we can address the impossibility of CRISPR/Cas9 to attack all the cells within the targeted pool? This work presents an alternative method for engagement of cell death in cancer cells with immediate application in the preclinical sector and significant translational relevance toward clinics.

## Main Text

Cancer is one of the most discussed subjects in experimental research today, with new genome-editing perspectives focused on CRISPR/Cas9. We witnessed some great preliminary results with the *ex vivo* approach, where the circulating cells were actually collected from the patients, modified with the editing tool, and then administered back into the patients. These approaches mainly involve the genetic manipulation of immune cells.^1^, ^2^, ^3^ But how do we deal with the systemic administration of CRISPR/Cas9 into patients? Two major issues are under experimental debate: (1) how we can hide this tool from the immune system recognition and deliver it into targeted cells and (2) how to obtain a general inhibitory effect upon the malignant mass in the absence of a possibility to target every cell within the tumor. In our previous article, which addressed the problems associated with *in vivo* delivery of CRISPR/Cas9, we stressed the necessity of hybrid ideas that can take advantage of the already existing resources to shorten the gap between preclinical and clinical trials.^4^

### Cancer-Engineered Exosomes and Necroptosis Activation via CRISPR/Cas9

If we look closer at the malignant entity behavior, we can see that the pathophysiological processes around it offer numerous possibilities that can be exploited against the tumor. This is the case of exosomes, small vesicles secreted by both normal and pathological cells to support communication with the microenvironment and sustain, at the same time, their phenotypical characteristics.^5^ Therefore, cancer cells intensely secrete these vehicles, which have a preferential tropism for close or distant malignant cells, with the purpose of sustaining their tumor microenvironment or even to prepare the metastatic niche.^5^, ^6^ A closer look at these nanovehicles raised the idea that exosomes can actually be used as natural delivery platforms for multiple forms of anti-cancer therapeutics like drugs, non-coding RNAs, or polyphenolic compounds.^7^, ^8^ Therefore, exosomes offer the possibility of delivering CRISPR/Cas9 in a free immunogenic manner by enclosing this editing tool in a stable environment encapsulated by a double lipid membrane naturally found in the circulatory system.^6^ This encapsulation became even more important after the discovery that we actually hold a pre-existing adaptive immunity against proteins like Cas9 that could easily eradicate the editing system within the organisms.^9^ But equally so, what will happen to the tumor cells that are not targeted by these engineered exosomes? The ability of the malignant accumulation to proliferate in an accelerated manner counteracts the effects of any strategy that allows the survival of a small percent of cancer cells, especially in advanced stages of the disease.

Initially, necrosis was considered an unorganized type of cell death not sustained by specific signaling mechanisms. However, recent research has proposed necroptosis as a form of programmed cell death that takes place under strict control by specific molecules encountered in the pathway. More specifically, the activation of TNFR1 by tumor necrosis factor (TNF)-alpha ligand determines the formation of the death complex, where cIAP1/2 plays a key role. If this molecule is active and directing the K63-linked polyubiquitination of receptor-interacting protein kinase member1 (RIP1), which translates downstream in activation of nuclear factor κB (NF-κB) and mitogen-activated protein kinase (MAPK) survival pathways, the cell remains viable. If IAP1/2 is not functional and Caspase 8 is active, the cytosolic complex RIP1, FADD, and Caspase-8 is formed, which is conducive to apoptosis.^10^ These pathways were consistently studied in experimental strategies for cancer therapy, but there is a possibility that, most of the time, it was overlooked. If IAP1/2 and Caspase-8 are inactive, a second complex is formed: RIP1, RIP3, and mixed lineage kinase domain like pseudokinase (MLKL), and the cell is forced to undergo necroptosis, a form of programmed necrosis.^10^ The advantage of this last form of programmed cell death consist in the immediate events after the installation of necroptosis, where the membrane is practically ruptured and the content of the cell is released in the microenvironment. This exposure actually liberates tumor-specific antigens that can be recognized by immature dendritic cells and conclude with the activation of T cells and specific attacking of surviving malignant entities.^11^ Therefore, even if apoptosis is now considered the “to go” pathway when trying to eliminate cancer cells, the cells that are actually forced to undergo apoptosis in the lack of a feedback possibility have minimal effects upon the neighbor ones. In the case of necroptosis, the death of one cell condemns the other ones by activating the immune system.

### Therapeutic Strategy

**Figure 1:**
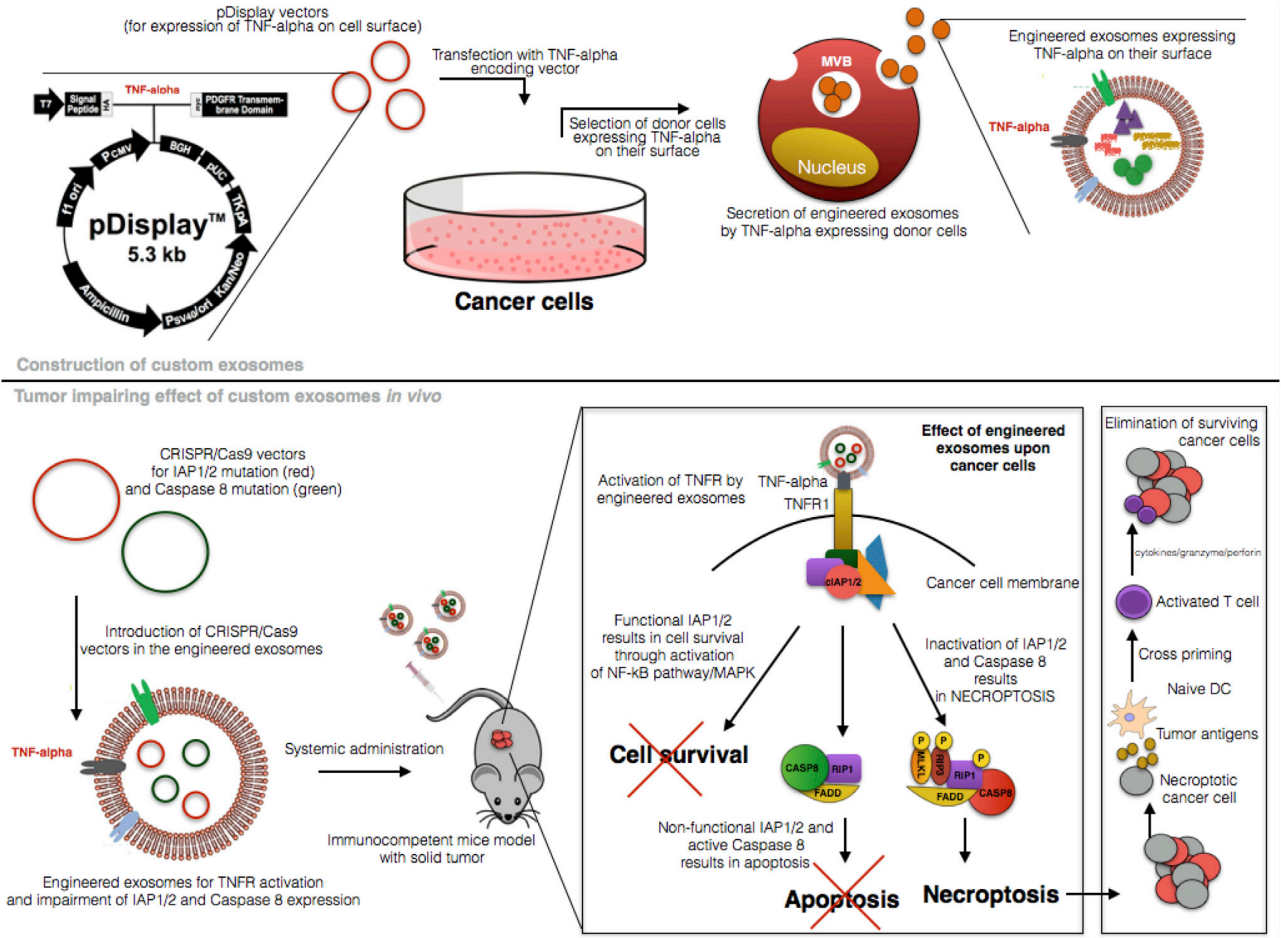
Schematic Representation of the Therapeutic Strategy Involving Activation of Necroptosis in Cancer and Induction of Cell Death Concomitant with Specific Activation of the Immune System against Cancer Cells

Therefore, the proposed strategy embraces a novel method for CRISPR/Cas9 delivery via exosomes. Exosomes can be engineered to preferentially target the malignant cells and, at the same time, start the necroptosis pathway. The enclosed message can take over once the editing tool is delivered into cells, where two simple CRISPR/Cas9 vectors will target IAP1/2 and Caspase 8 (Figure 1). In the next paragraphs we will present the anti-cancer strategy step-by-step.

The first step within this proposal consists of activation of TNFR1 (TNFRSF1A) by TNF-alpha ligand to further activate the death-associated pathways. Cancer cells (that secrete exosomes at a higher rate than healthy cells due to an increased metabolism and a “desire” to maintain the malignant environment) will be genetically engineered to express TNF-alpha on their surface and then secrete exosomes that mimic the same pattern: exosomes with TNF-alpha protein enclosed in the double lipid membrane. This step can be achieved by transfecting the tumor cells with a pDisplay vector that will anchor TNF-alpha to the cell surface by cloning the gene of interest in frame with the vectors unique to the N-terminal secretion signal and the C-terminal transmembrane anchoring domain of platelet-derived growth factor receptor (PDGFR). Ohno et al.^12^ used a similar approach, where they cast-off this strategy to obtain secreted exosomes with GE11 peptide on their surface, a ligand for EGFR that is abundantly expressed by tumor cells (breast cancer cells). They further used these exosomes for let-7a miRNA delivery, a sequence with tumor suppressor effects on cancer cells.^12^ This study and other similar ones sustain the possibility of engineering exosomes with the final purpose of including artificial proteins in their membrane. Once these exosomes are produced and selected with specific antibodies for TNF-alpha (designed to be expressed on their surface), they will be used as a delivery platform for two simple CRISPR/Cas9 vectors targeting two important molecules within the necroptotic pathway: IAP1/2 and Caspase 8. We previously affirmed that, in the absence of functional IAP1/2 and Caspase 8, the cell is forced to undergo necroptosis, a form of programmed cell death with further stimulatory effects upon the immune system. The simple nature of the genomic editing vectors, mainly comprised of only a single gRNA for IAP1/2 and Caspase 8 and Cas9 sequence, is actually a crucial factor, where the limited space within the exosomes can be used at maximum capacity. Moreover, we are aiming to introduce a specific promoter for Cas9, PEG promoter, which was previously shown to function only in cancer cells and is non-active in healthy ones.^13^ This is an important regulatory factor, considering that, in a small percent, exosomes derived from cancer cells also could target healthy ones despite the increased tropism for malignant entities. Once the two CRISPR/Cas9 vectors are loaded into the selected exosomes by electroporation, the therapeutic tool can be tested *in vivo* through systemic administration into immunocompetent humanized mice models bearing malignant tumors with metastatic potential. Once the exosomes reach their target (malignant cells) and induce necroptosis in the transfected ones, the immune system can be activated by the release of tumor-specific antigens able to specifically engage naive dendritic cells. These last ones will further mature and expand into activated dendritic cells with the possibility of engaging through cross-priming naive CD8+ cells. Cytotoxic T cells originating from differentiation of naive CD8+ cells will infiltrate the tumor to eradicate surviving malignant cells^11^ that were previously not targeted by the engineered exosomes. By this strategy, we could not just offer a stable delivery vehicle for CRISPR/Cas9, but we also could engage the tumor cells into a suicidal pathway that will propagate toward elimination of the malignant mass.

### Future Directions

The present strategy offers the possibility of using CRISPR/Cas9 (a tool with immense potential in cancer treatment) through *in vivo* delivery to eradicate solid tumors. This could represent only the beginning of these types of strategies, where numerous other adjustments can be made in terms of exosome specificity and CRISPR/Cas9-targeted sequences. Moreover, the current proposal can be applied to different types of malignancies, ideally those that have a high level of TNFR1, but not being limited to a specific one. We are aware that the proposed stratagem can have limitation factors like over-activation of the immune system, decreased specificity of exosomes, and limited expression of TNFR1 in several types of cancer. However, this proposal is a possible new and efficient method for *in vivo* CRISPR/Cas9 delivery and abolition of solid tumors.

### Screening of Solid Malignancies for the Proposed Strategy: Expression of Tnfrsf1a and TNF-alpha

Recently, CRISPR/Cas9 wide screening of mouse genes involved in necroptosis identified Tnfrsf1a, a gene encoding for a TNF receptor, as one of the genes associated with the most significant single guide RNA (sgRNA) enrichment.^14^ The strong implication of Tnfrsf1a in activation of the necroptosis pathway was also previously shown by Hitomi et al.^15^ in a similar study.

**Figure 2:**
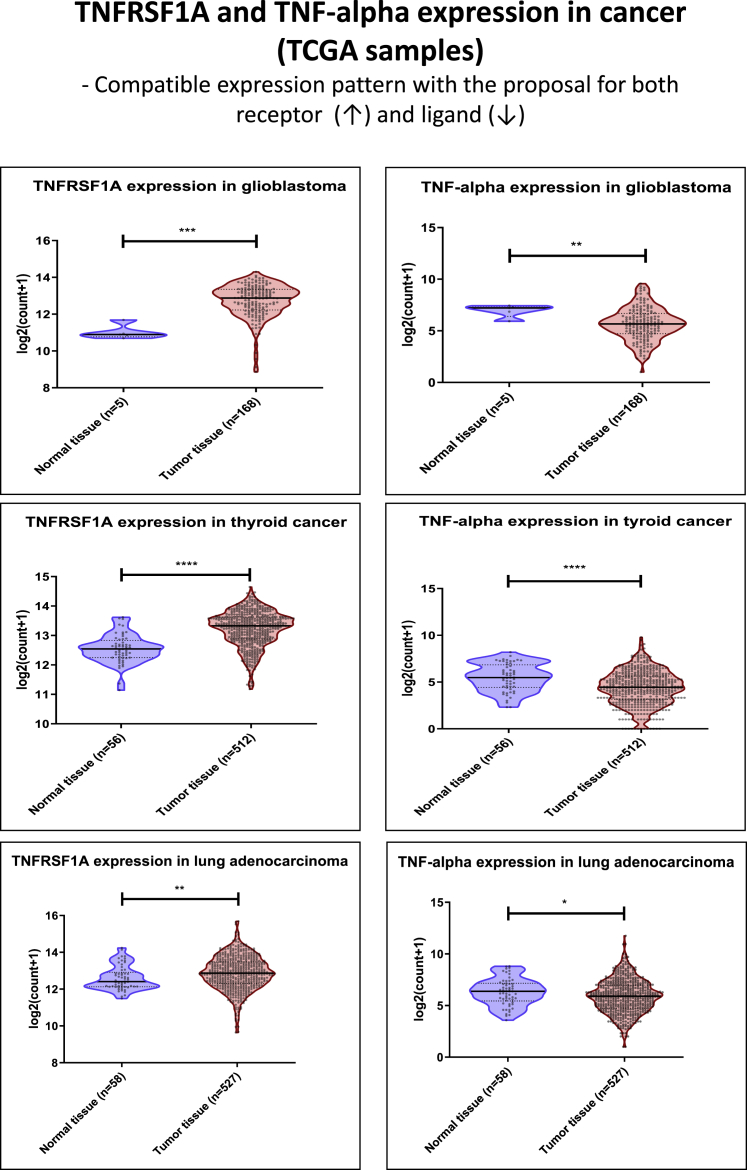
TCGA RNA-Seq Data Screening for TNFRSF1A and TNF-alpha Expression in Cancer Compatible expression with the proposed strategy (glioblastoma, thyroid cancer, and lung adenocarcinoma).

Specific activation of the programmed necrosis pathway by the TNF-alpha ligand expressed in the membrane of the cancer-derived exosomes is an essential part of the present proposal. Therefore, we screened different types of solid cancers for Tnfrsf1a and TNF-alpha endogenous expression by analyzing RNA sequencing (RNA-seq) data from The Cancer Genome Atlas (TCGA) samples. Screening is important for possible identification of malignancies with Tnfrsf1a overexpression compared with controls, which will confer increased specificity of the engineered exosomes for tumor cells. TNF-alpha screening is also essential for identification of potential competitiveness between the ligand expressed in the exosomes’ membrane and the endogenous molecules. We screened 18 cancer types and/or subtypes (Figures 2 and S1–S3) in terms of Tnfrsf1a and TNF-alpha expression.

All types of screened malignancy, glioblastoma, thyroid cancer, and lung adenocarcinoma (Figure 2) showed compatible data with our proposal, where Tnfrsf1a receptor is upregulated and TNF-alpha ligand is downregulated at the transcript level. Other types of cancer showed opposite results in terms of TNF-alpha expression, where esophageal, head and neck (Figure S1), stomach and endometrioid cancer, and sarcoma (Figure S3) presented significantly increased levels of the ligand, which suggests an oncogenic role. Other malignancies showed reduced expression for TNF-alpha (pancreatic, prostate, liver and colon cancer, and lung squamous cell carcinoma) (Figure S2), but not increased expression of the receptor.

## Author contributions

I.B.-N. and D.G. conceptualized and designed the proposal; D.G. wrote the paper, and I.B.-N. revised it before submission.

## Conflicts of interest

The authors declare no competing interests.

## Supplemental Information

Document S1. Figures S1–S3

Document S2. Article plus Supplemental Information
