# Supplementary material for: Activation of Necroptosis by Engineered Self Tumor-Derived Exosomes Loaded with CRISPR/Cas9
Source: Mol Ther Nucleic Acids. 2019 Jul 3;17:448–51. doi: 10.1016/j.omtn.2019.05.032 (PMC6639591; doi:10.1016/j.omtn.2019.05.032)
Supplement: Document S1. Figures S1–S3 [file mmc1.pdf]

OMTN, Volume *17*

Supplemental Information

Activation of Necroptosis by Engineered Self Tumor-Derived Exosomes Loaded with CRISPR/Cas9

Diana Gulei and Ioana Berindan-Neagoe

TNFRSF1A and TNF-alpha expression in cancer (TCGA samples)

- Compatible expression pattern with the proposal for the receptor (↑), but not for the ligand (↑ or not statistically significant)

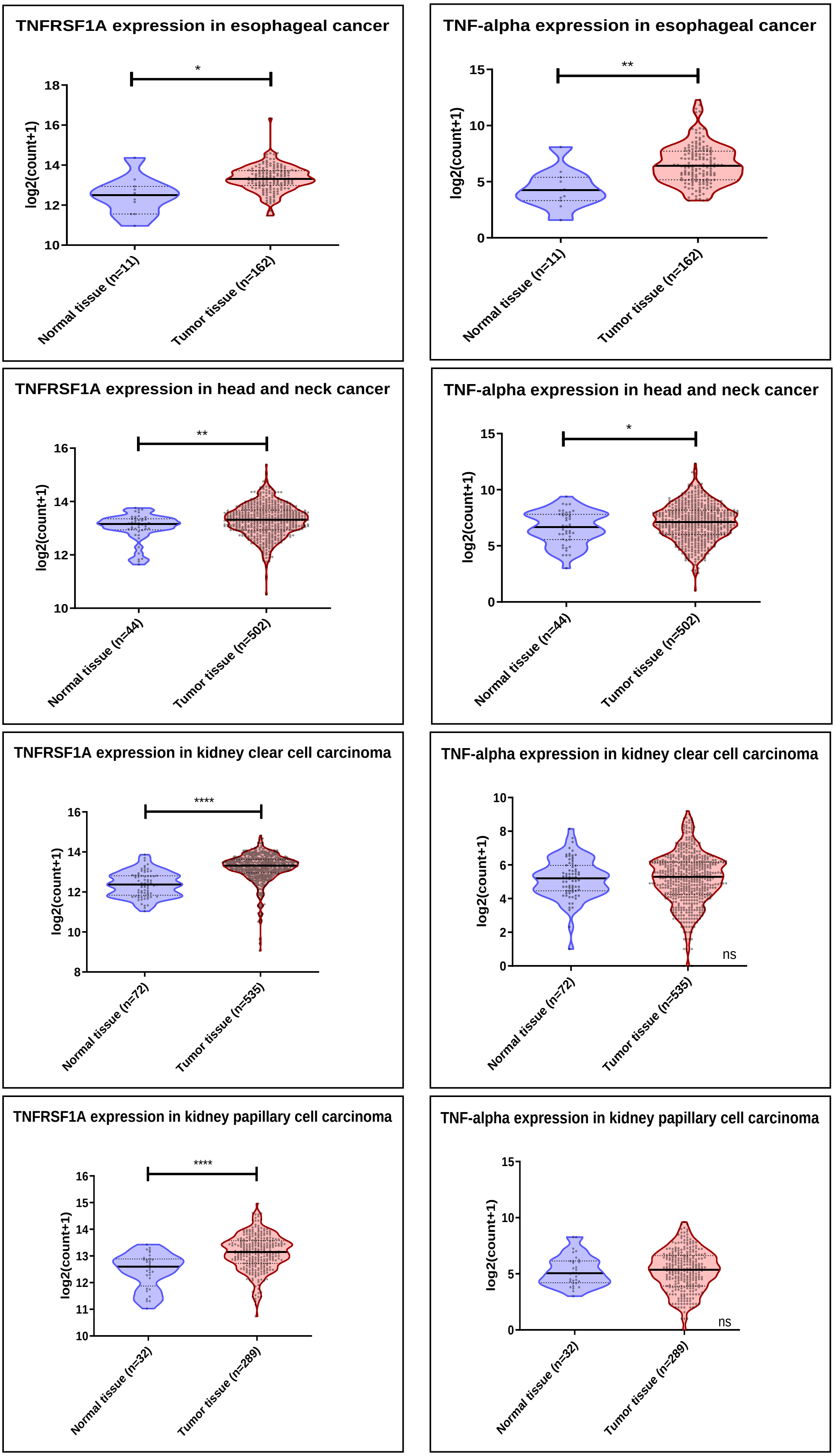

TNFRSF1A and TNF-alpha expression in cancer (TCGA samples)

- Compatible expression pattern with the proposal for the ligand (↓), but not for receptor (↓ or not statistically significant)

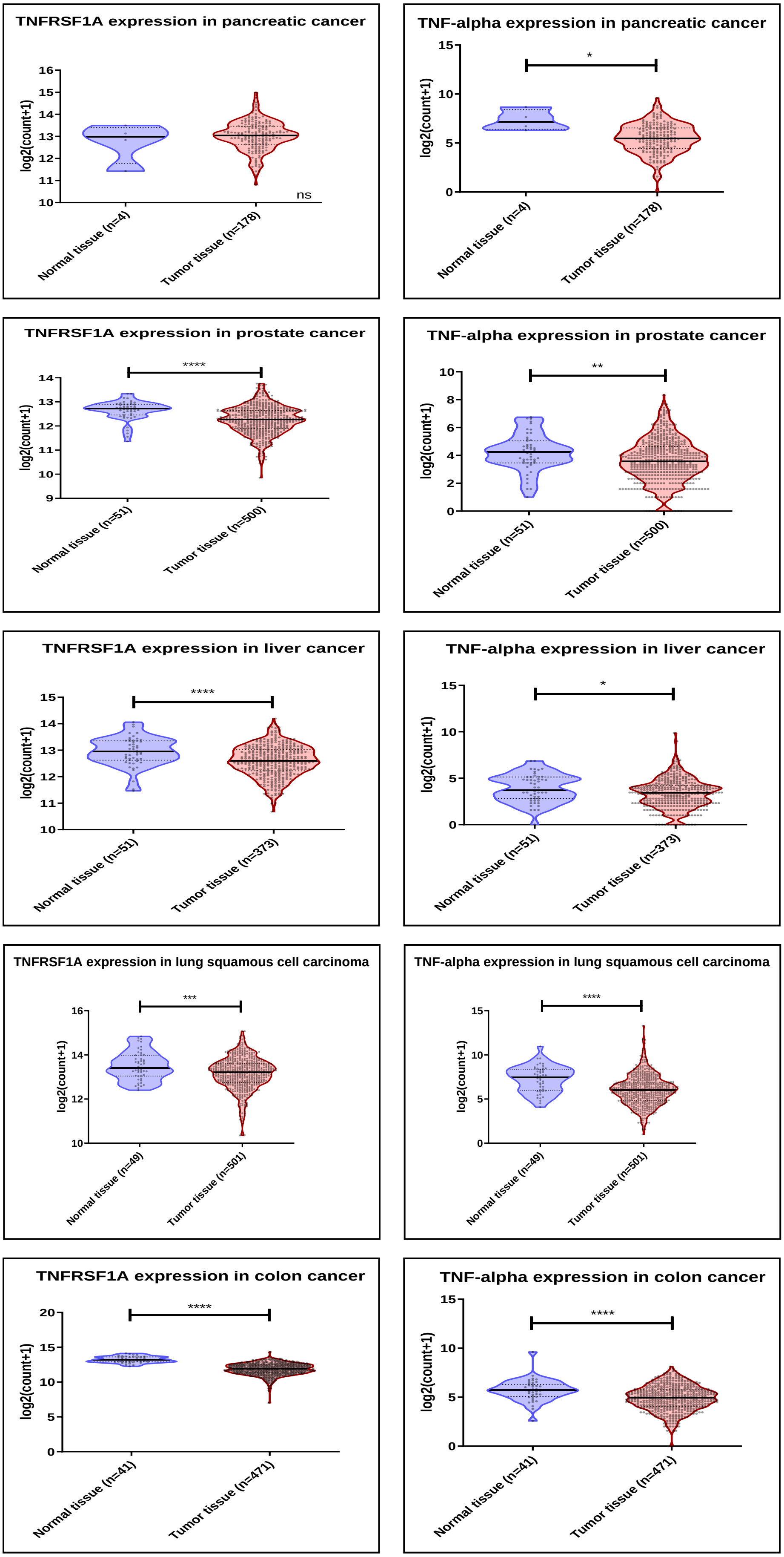

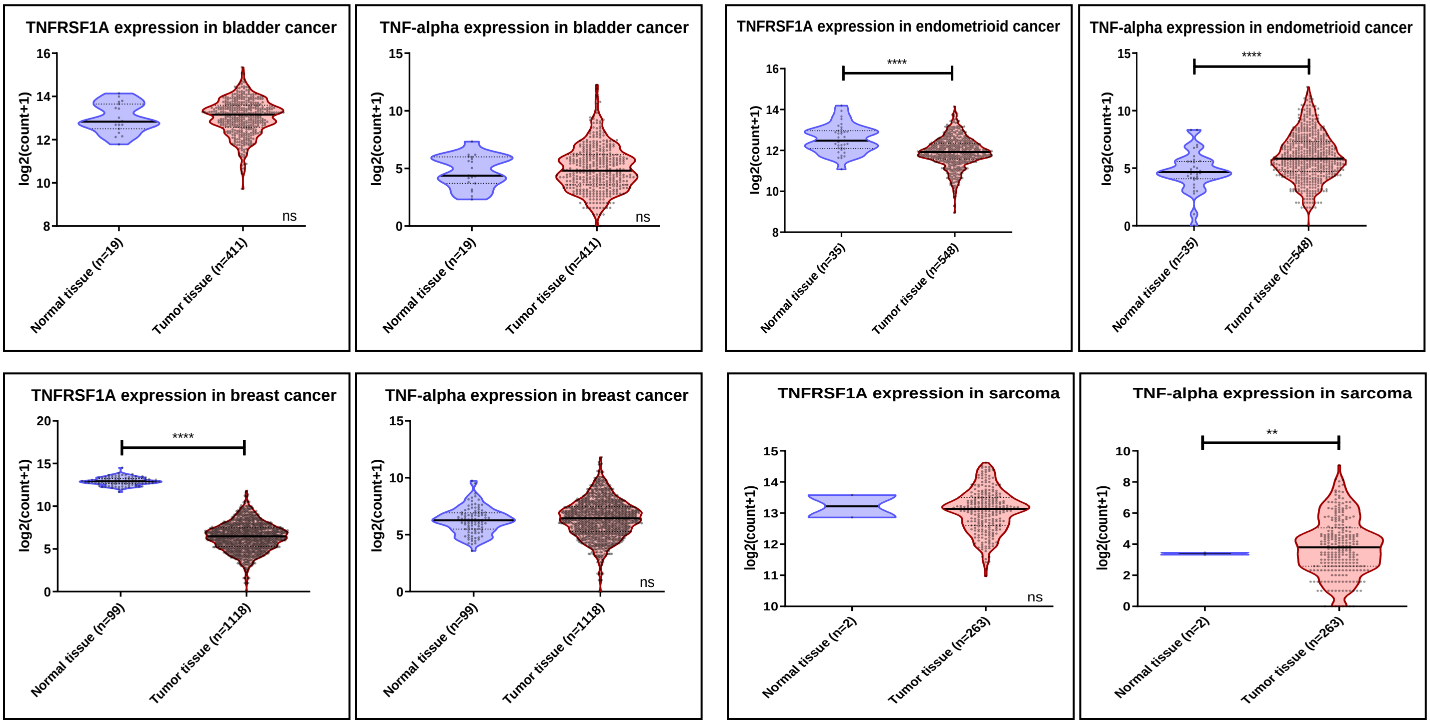

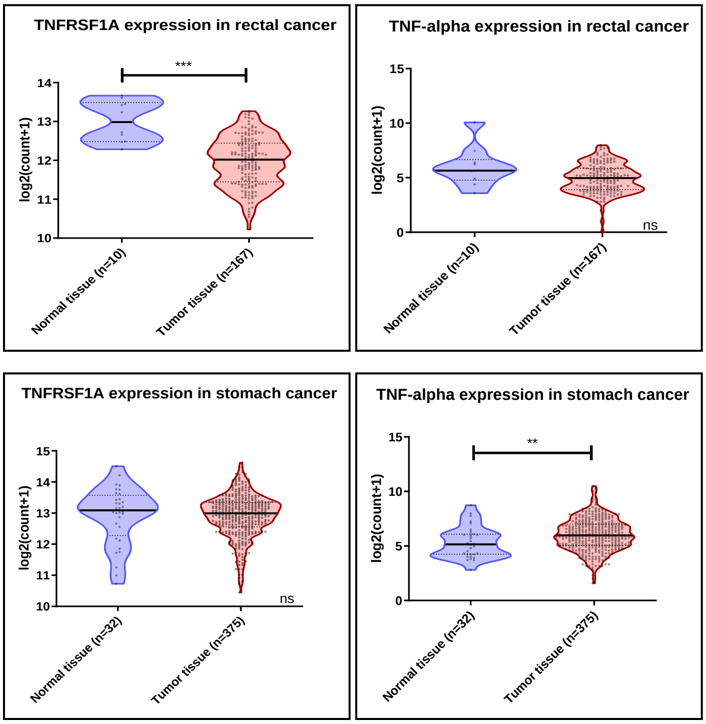

TNFRSF1A and TNF-alpha expression in cancer (TCGA samples)

- Expression pattern not compatible with the proposed strategy - receptor (↓ or not statistically significant) and ligand (↑ or not statistically significant)
